# A Case of Chronic Expanding Hematoma Presenting as a Subcutaneous Mass 24 Years after Konno Procedure

**DOI:** 10.70352/scrj.cr.25-0310

**Published:** 2025-10-21

**Authors:** Yoshinobu Watabe, Koji Sato, Taiki Ito, Satoru Wakasa

**Affiliations:** 1 Department of Cardiovascular Surgery, KKR Sapporo Medical Center, Sapporo, Hokkaido, Japan; 2 Department of Cardiovascular Surgery, Sapporo Kojinkai Memorial Hospital, Sapporo, Hokkaido, Japan; 3 Department of Cardiovascular Surgery, Faculty of Medicine and Graduate School of Medicine, Hokkaido University, Sapporo, Hokkaido, Japan

**Keywords:** chronic expanding hematoma, Konno procedure, right ventricular outflow tract reconstruction

## Abstract

**INTRODUCTION:**

Here, we report a very rare case of chronic expanding hematoma presenting as a gradually enlarging subcutaneous pulsatile mass detected 24 years after a Konno procedure.

**CASE PRESENTATION:**

A 37-year-old man, who had undergone a Konno procedure at the age of 13 for severe aortic stenosis caused by a bicuspid aortic valve, presented with a pulsatile subcutaneous mass that had gradually increased in size over a 4-month period. CT revealed a large mediastinal mass compressing the right ventricular outflow tract, and a transthoracic echocardiogram showed an elevated pressure gradient between the right ventricle and pulmonary artery. Surgical resection of the mass and reconstruction of the right ventricular outflow tract were performed. The mass was completely excised, and the outflow tract was reconstructed using a short valved composite graft. The patient had an uneventful postoperative course.

**CONCLUSIONS:**

This case underscores the importance of considering chronic expanding hematoma in the differential diagnosis of new pulsatile masses in patients with a history of cardiac surgery and demonstrates that timely surgical intervention can be performed safely and effectively.

## Abbreviations

CEH
: chronic expanding hematoma
RVOT
: right ventricular outflow tract
TTE
: transthoracic echocardiogram

## INTRODUCTION

Chronic expanding hematoma (CEH), first described by Reid in 1980, refers to a hematoma that gradually enlarges for more than one month after the initial bleeding event.^1)^ CEH may occur following cardiac surgery, with reported intervals ranging from 1 to 30 years.^2)^ In such cases, CEH is most commonly detected due to symptoms of refractory heart failure caused by cardiac compression from an intrapericardial hematoma. Here, we report a very rare case of CEH presenting as a gradually enlarging subcutaneous pulsatile mass detected 24 years after a Konno procedure.

## CASE PRESENTATION

**Fig. 1:**
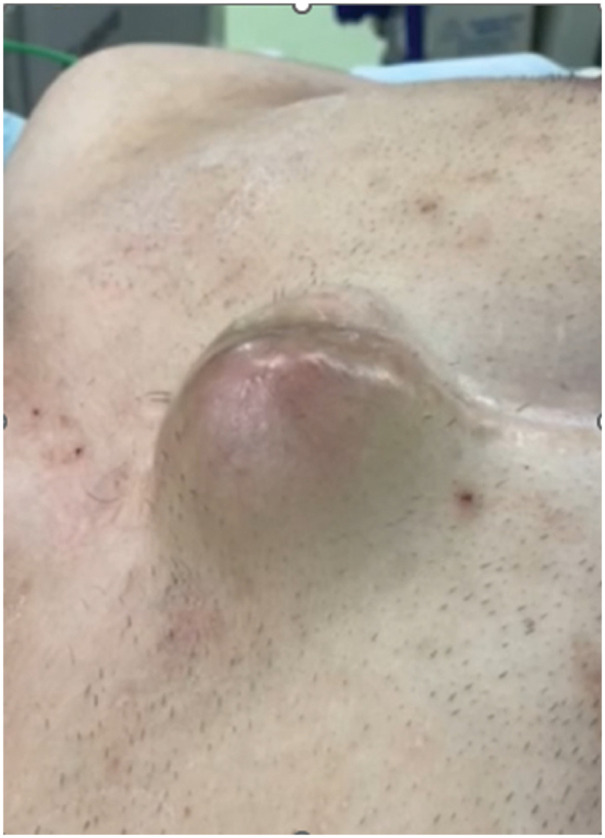
Clinical photograph showing a pulsatile subcutaneous mass on the anterior chest wall.

**Fig. 2:**
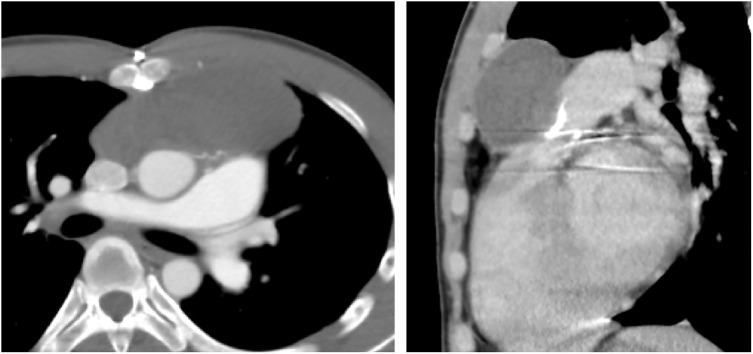
Axial and sagittal CT images obtained 11 years prior to the current presentation, showing a mediastinal mass measuring approximately 100 × 35 mm.

**Fig. 3:**
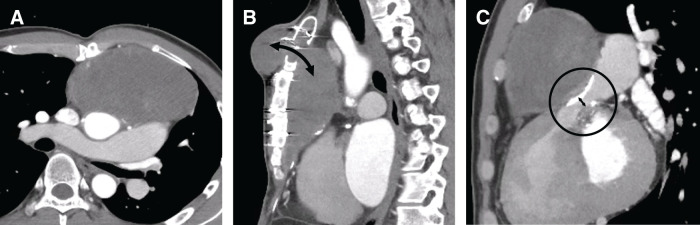
Preoperative CT images of chronic expanding hematoma. (**A**) Axial image demonstrating a mediastinal mass measuring 110 × 65 mm. (**B**) Sagittal image showing continuity between the subcutaneous and mediastinal masses through an osteolytic defect in the manubrium (black arrow). (**C**) The mediastinal mass compressing the right ventricular outflow tract (circle), with narrowing to 9 mm in diameter (double-headed arrow).

A 37-year-old man presented to our hospital with a pulsatile subcutaneous mass on the anterior chest wall, which was visibly pulsating in synchrony with the heartbeat and had gradually increased in size over a 4-month period (**Fig. 1**). He had been diagnosed with a congenital bicuspid aortic valve at the age of 3 and had undergone a Konno procedure with a 23-mm SJM valve (St. Jude Medical, St. Paul, MN, USA) for severe aortic stenosis at the age of 13. Eleven years earlier, CT had shown a mediastinal mass measuring approximately 100 × 35 mm, suggestive of a hematoma (**Fig. 2**), and transthoracic echocardiography (TTE) at that time revealed a right ventricular outflow tract (RVOT) pressure gradient of 47 mmHg. As he remained asymptomatic, no further imaging was performed until the current presentation. On the current admission, CT demonstrated a mediastinal mass measuring approximately 110 × 65 mm—showing no marked change in size—compressing the RVOT, indicating high internal pressure, and extending anteriorly into a subcutaneous mass through an osteolytic defect in the manubrium of the sternum (**Fig. 3**). There was no contrast enhancement within the mass, and TTE confirmed the absence of blood flow on color Doppler imaging. The RVOT pressure gradient had increased to 59 mmHg, and the tricuspid regurgitation pressure gradient was 87 mmHg. Moderate tricuspid regurgitation was observed, with an enlarged tricuspid annulus measuring 42 mm in diameter. The patient had no symptoms of heart failure. Preoperative blood tests showed no evidence of systemic inflammation, with a white blood cell count of 7200/μL and a C-reactive protein level of 0.11 mg/dL.

Surgery was performed for hematoma excision and hemostasis, based on the suspicion that blood oozing from the suture line of the RVOT patch (equine pericardium reinforced with a Dacron [polyethylene terephthalate] patch; Vascutek Terumo, Scotland, UK) was the cause of the hematoma. Tricuspid annuloplasty and RVOT reconstruction were also planned, if necessary. Prior to median resternotomy, the bilateral femoral arteries and right femoral vein were cannulated to allow for rapid initiation of cardiopulmonary bypass in the event of massive bleeding.

After systemic heparinization, the skin and subcutaneous mass were incised, releasing brownish-red exudates without evidence of active bleeding. A median resternotomy was then performed. The sternum was found to be partially deficient, consistent with the area of hematoma extension. The mediastinum was filled with organized hematoma. After evacuation of the hematoma and copious saline irrigation, oozing was primarily observed from the suture line of the RVOT patch adjacent to the pulmonary valve. The bleeding was dark red and non-pulsatile, consistent with chronic low-pressure leakage rather than active arterial hemorrhage. No disruption or cracking of the patch material was seen. Cardiopulmonary bypass was established via bilateral femoral artery perfusion and right femoral vein drainage. A longitudinal incision was made from the pulmonary artery trunk through the anterior wall of the RVOT patch, extending partially into the anterior wall of the right ventricle. The RVOT patch was located near the pulmonary valve, necessitating pulmonary valve replacement.

Due to severe calcification on the posterior wall of the RVOT, the RVOT was reconstructed using the Lantern procedure with a composite graft.^3)^ The graft consisted of a 27-mm bioprosthetic valve (Epic Supra; Abbott Vascular, Chicago IL, USA) and a 30-mm prosthetic vascular graft (J-graft; Japan Lifeline, Tokyo, Japan). The composite graft was anastomosed to the most distal segment of the pulmonary trunk feasible, using a 4-0 polypropylene suture. It was then covered with a prosthetic graft patch, which was anastomosed to the remnant portion of the previous RVOT patch and the right ventricular wall to reconstruct the anterior wall of the new RVOT. Tricuspid annuloplasty was also performed using a 28-mm prosthetic ring (Contour 3D; Medtronic, Minneapolis, MN, USA). Weaning from cardiopulmonary bypass was uneventful. The sternum was closed with sternal wires, and the skin was sutured in the standard fashion. Intraoperative Gram staining of the hematoma content revealed numerous white blood cells but no bacteria, and bacterial cultures were also negative, indicating no evidence of active infection.

**Fig. 4:**
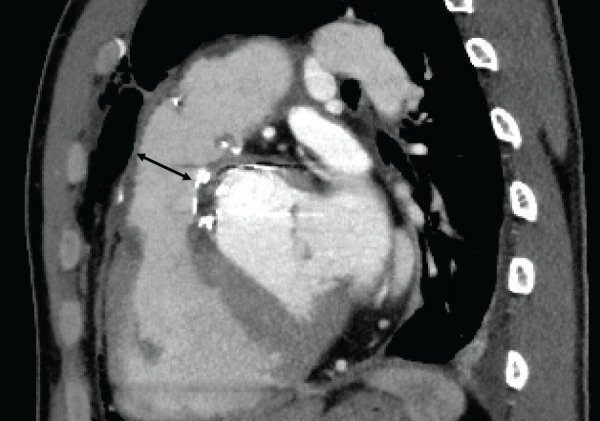
Postoperative CT image showing relief of RVOT stenosis, with the RVOT diameter increased to 22 mm (double-headed arrow). RVOT, right ventricular outflow tract

Postoperative CT demonstrated relief of the RVOT stenosis (**Fig. 4**). TTE and the right heart catheterization showed that the pressure gradient between RVOT and pulmonary artery had decreased from 59 to 17 mmHg. The patient’s postoperative course was uneventful, and he was discharged on POD 14.

## DISCUSSION

CEH could develop in various regions of the body. In cases involving the pericardial cavity, several reports have described CEH following cardiac surgery, trauma, or epicardial injury.^2,4–7)^ Although the exact mechanism remains unclear, previous studies have suggested multiple contributing factors, including persistent oozing from the suture line, impaired absorptive capacity of the pericardium, and inflammatory responses associated with the breakdown products of the hematoma itself.^8,9)^ Among reported cases of CEH after cardiac surgery, most were detected within 10–15 years postoperatively. The longest interval described in the literature is 30 years after tetralogy of Fallot repair,^2)^ and cases occurring more than 20 years postoperatively are rare. No previous reports have described CEH presenting as a subcutaneous mass.

In the present case, the hematoma was presumed to have originated from chronic blood leakage at the anastomotic site of the previous Konno procedure. Neither contrast-enhanced CT nor transthoracic echocardiography showed intramass blood flow, making a pseudoaneurysm unlikely. While most previously reported cases of CEH were diagnosed due to symptoms of heart failure resulting from mediastinal compression,^2)^ this patient remained asymptomatic and was diagnosed after the detection of a gradually enlarging subcutaneous mass. This atypical presentation might have been due to the slow progression of hematoma formation, which allowed the patient to physiologically adapt to elevated right ventricular pressure.

In this case, severe calcification of the previous RVOT patch likely increased tissue fragility at the suture line and made the site more susceptible to pulsatile stress. We speculate that a small initial bleed at this weakened site led to persistent leakage and the formation of a hematoma. This hematoma gradually enlarged and compressed the RVOT, elevating right ventricular pressure. The increased pressure at the bleeding point, in turn, promoted further leakage, creating a vicious cycle that drove the slow expansion of the hematoma over many years. Preoperative CT showed an osteolytic defect in the manubrium continuous with the mediastinal and subcutaneous mass, whereas no such defect was present 11 years earlier. This suggests gradual sternal erosion from chronic hematoma compression, which eventually created a communication into the subcutaneous space. Once this occurred, the reduced anatomical resistance allowed intralesional pressure to be transmitted directly to the subcutaneous component, leading to its rapid enlargement in the 4 months before surgery.

In most cases, definitive diagnosis of CEH requires surgical resection and histopathological evaluation,^4)^ and complete removal of the hematoma along with control of the bleeding source remains the cornerstone of treatment. Due to the difficulty in differentiating CEH from pseudoaneurysm using preoperative imaging modalities, there is a risk of unexpected massive bleeding upon incision. Although such events are relatively uncommon, they may be life-threatening when they occur. Therefore, preparation for rapid initiation of cardiopulmonary bypass is essential, and secure arterial and venous access should be considered a critical component of preoperative planning.

In cases with a long interval after the initial operation, marked calcification of previously manipulated cardiac structures may be encountered. In the present case, severe calcification of the posterior RVOT wall precluded direct anastomosis. To address this, a Lantern procedure—previously reported by the authors—was employed, which enabled secure placement of a prosthetic pulmonary valve without requiring anastomosis to the heavily calcified tissue.^3)^

## CONCLUSIONS

We described a rare case of CEH presenting as a subcutaneous mass 24 years after a Konno procedure. Although uncommon, CEH should be considered in the differential diagnosis of progressively enlarging chest wall masses in patients with a remote history of cardiac surgery. Furthermore, for patients with a history of RVOT reconstruction, regular long-term imaging follow-up—such as echocardiography or CT—should be considered to allow earlier detection of rare but potentially serious late complications, including CEH.
